# Scanning electrochemical microscopy studies of micropatterned copper sulfide (Cu_*x*_S) thin films fabricated by a wet chemistry method

**DOI:** 10.1016/j.electacta.2011.03.100

**Published:** 2011-05-30

**Authors:** Miao Chen, Jing Zhao, Xiaocui Zhao

**Affiliations:** CSIRO Minerals, Clayton, Victoria, 3168, Australia

**Keywords:** Copper sulfide (Cu_*x*_S), Micro-pattern, Self-assembled monolayer (SAM), Scanning electrochemical microscopy (SECM)

## Abstract

Patterned copper sulfide (Cu_*x*_S) microstructures on Si (1 1 1) wafers were successfully fabricated by a relatively simple solution growth method using copper sulfate, ethylenediaminetetraacetate and sodium thiosulfate aqueous solutions as precursors. The Cu_*x*_S particles were selectively deposited on a patterned self-assembled monolayer of 3-aminopropyltriethoxysilane regions created by photolithography. To obtain high quality Cu_*x*_S films, preparative conditions such as concentration, proportion, pH and temperature of the precursor solutions were optimized. Various techniques such as optical microscopy, atomic force microscopy (AFM), X-ray diffraction, optical absorption and scanning electrochemical microscopy (SECM) were employed to examine the topography and properties of the micro-patterned Cu_*x*_S films. Optical microscopy and AFM results indicated that the Cu_*x*_S micro-pattern possessed high selectivity and clear edge resolution. From combined X-ray diffraction analysis and optical band gap calculations we conclude that Cu_9_S_5_ (digenite) was the main phase within the resultant Cu_*x*_S film. Both SECM image and cyclic voltammograms confirmed that the Cu_*x*_S film had good electrical conductivity. Moreover, from SECM approach curve analysis, the apparent electron-transfer rate constant (*k*) in the micro-pattern of Cu_*x*_S dominated surface was estimated as 0.04 cm/s. The SECM current map showed high edge acuity of the micro-patterned Cu_*x*_S.

## Highlights

► Copper sulfide (Cu_*x*_S) microstructures on Si wafer has been prepared using a wet chemistry method. ► To obtain high quality Cu_*x*_S films, preparative conditions were optimized. Scanning electrochemical microscopy (SECM) was employed to examine the properties of the Cu_*x*_S films. ► The apparent electron-transfer rate constant (k) of Cu_*x*_S surface was estimated as 0.04 cm/s. ► The SECM current map showed high edge acuity of the micro-patterned Cu_*x*_S.

## 1 Introduction

Copper sulfide (Cu_*x*_S, 1 ≤ *x* ≤ 2) thin films have been found to have very useful electrical and optical properties and have attracted great interest for their potential use in energy applications, such as applications in achievement of solar cells and in photochemical conversion of solar energy as solar absorber coating [1,2], as selective radiation filters on architectural windows for solar control in warm climates [3], and as electroconductive coatings deposited on organic polymers [4]. At room temperature, there are five stable copper sulfide phases. Copper-rich phases exist as chalcocite (Cu_2_S), djurlite (Cu_1.95_S), digenite (Cu_1.8_S) and anilite (Cu_1.75_S), while the sulfur-rich phase exists as covellite (CuS). Mixed phases with intermediate compositions are also known. Copper sulfide (Cu_*x*_S) usually exhibits semi-metallic properties, intrinsic semi-conductivities and in some cases, ductility [5–7]. It forms a p-type transparent film, and although transparent p-type materials generally have inferior conductivities to the widely used transparent n-type conducting materials such as ITO (In_2_O_3_:Sn) [8], FTO (SnO_2_:F) [9], etc. [10,11], we show here that Cu_*x*_S has semi-metallic conductivity.

Previous studies have mainly focused on the preparation of bulk Cu_*x*_S film and numerous techniques have been investigated, such as vacuum evaporation [12], activated reactive evaporation [13], reactive magnetron sputtering [14], spray pyrolysis [15,16], slurry technique [17], and chemical bath deposition [18]. The sulfur source agents reported included thiourea, sodium sulfide and sodium thiosulfate, while copper ions were complexed by triethanolamine, ammonia, ethylenediaminetetraacetate (EDTA), citric acid or 1,4,8,11-tetrazacyclo-tetradecane. However, the production of patterned Cu_*x*_S microstructure on a solid surface has not been reported. Patterning surfaces leads to the creation of physicochemical heterogeneities (e.g. surface energy, chemical reactivity, conductivity, topography and so forth), which are an important issue in the design of complex components used in high-tech technologies [19]. Furthermore, the creation of micro-patterned semiconductor materials has become an important subject in many areas, including microfluidics, optics, biosensors, electronics, and information storage [20–23].

A number of techniques have been successfully demonstrated for making patterned micro-features on various substrates, such as deposition by scanning electrochemical microscopy, screen printing, micromolding in capillaries (MIMIC), photolithography, laser writing, surface-templated deposition, microcontact printing and so on [24–28]. Among these methods, photolithography is one of the best-established technologies for micro-patterning and has found wide applications in the microelectronics industry because of advantages such as large-scale production and simple processing [29]. In this method, the pattern is produced by exposing a thin film of photoresist, light-sensitive polymer, or self-assembled monolayer (SAM) to UV light through a photomask. Either the patterned photoresist film or SAM has been used as a template for the subsequent deposition or etching. Some semiconductor microstructures have been prepared by photogenerated carriers without using photoresist or SAM, as reported in our previous study [30]. 3-Aminopropyltriethoxysilane (APTES) is a silane coupling agent which has been commonly used to provide surface protection. APTES is comprised of two reactive functional groups. The amino tail is able to form a strong covalent bond to a variety of metals such as Ag, Au and Cu. The ethoxy headgroup (–OC_2_H_5_) can undergo hydrolysis and bind to surface groups such as silanols on oxidized silicon.

In the present work, we report preliminary results on the patterning of Cu_*x*_S films, which combines UV-photolithography and area-selective deposition. It provides a convenient, low-cost, stable method for forming patterned microstructures of copper sulfide semiconductor on solid substrates. The morphology and structure of the resultant Cu_*x*_S patterning were characterized by optical microscopy, atomic force microscopy (AFM), and X-ray diffraction. Optical absorption and scanning electrochemical microscopy (SECM) were employed to investigate the corresponding optical band gap and electrochemical properties.

## 2 Simulation

The COMSOL multiphysics software (version 3.4) was employed to investigate the electrical property of the synthesized Cu_*x*_S substrate by simulating the probe approach curves (PACs) above the Cu_*x*_S substrate [31], which is similar to the work done by Cornut and Lefrou who reported an infinite substrate in the simulation model [32]. The redox mediator, ferrocenemethanol (FcMeOH), can be oxidized to FcMeOH^+^ at the surface of ultramicroelectrode (UME) (radius of *a*) which is sealed in glass capillary (Rg radius) and positioned above the substrate with a distance of *d* at a biased potential of 0.4 V *vs.* Ag/AgCl:

(1) $$\text{FcMeOH}-{\text{e}}^{-}\to {\text{FcMeOH}}^{+}$$

**Fig. 1:**
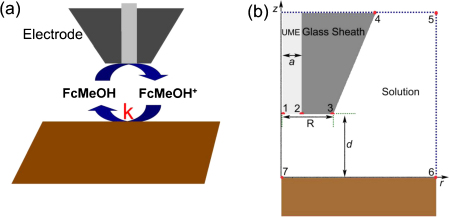
(a) Diagram of SECM feedback mode on Cu_*x*_S substrate and (b) geometry of simulation model.

FcMeOH^+^ can be reduced back to FcMeOH at the Cu_*x*_S substrate with the apparent electron-transfer rate constant of *k* (Fig. 1a):

(2) $${\text{FcMeOH}}^{+}+{\text{e}}^{-}\overset{k}{⟶}\text{FcMeOH}$$

FcMeOH^+^ generated at the electrode surface can diffuse through the solution and was reduced at Cu_*x*_S substrate (the line between points 6 and 7 in Fig. 1b) under the kinetic control. The flux of FcMeOH can be expressed as [33]:

(3) $$D{\left[\frac{\partial c(r,z)}{\partial z}\right]}_{z=-d}=k[c_{0}-c(r,-d)]$$

where *D* is the diffusion coefficient of FcMeOH (7 × 10^−10^ m^2^/s) [34], *r* and *z* are the coordinates shown in Fig. 1, *c*(*r*, −*d*) is the local concentration of FcMeOH and *c*_0_ is the bulk concentration of FcMeOH (0.9 mol/m^3^). Other simulation conditions can be found in the supporting information. A series of approach curves is calculated for fixed values of *k* by computing normalized current at 22 discrete normalized tip-substrate separation distances. The value of *k* at Cu_*x*_S substrate was obtained from the simulated PAC that overlapped the experimental one.

## 3 Experimental

### 3.1 Materials

The substrates were (1 1 1)-oriented single crystal silicon wafers, with a thickness of about 0.5 mm and a diameter of 125 mm, purchased from GRINM Semiconductor Materials Co. Ltd., Beijing. The as-received wafers, polished on one side and doped as n-type, were cut to a size of 2 cm × 2 cm. 3-Aminopropyltriethoxysilane (APTES, 99%) was purchased from Aldrich (USA) and used as-received. CuSO_4_·5H_2_O (Fluka, USA), Na_2_S_2_O_3_·5H_2_O (Sigma–Aldrich, USA), EDTA (Sigma–Aldrich, USA), H_2_SO_4_ (Sigma–Aldrich, USA), H_2_O_2_ (Merck, Germany), KNO_3_ (Sigma–Aldrich, USA) and ferrocenemethanol (FcMeOH) (Aldrich, USA) were used directly without further purification.

### 3.2 Preparation of patterned SAM

The silicon wafers were first cleaned by ultrasonicating in acetone, then in Milli-Q water, and then immersed in freshly prepared piranha solution (a mixture of 7:3 (v/v) 98% H_2_SO_4_ and 30% H_2_O_2_) at 90 °C for 1 h to remove organic residues and complete hydroxylation (*Caution: piranha solution reacts violently with many organic compounds and should be handled very carefully!*). The wafers were then rinsed thoroughly with Milli-Q water, placed into the APTES solution of 5.0 × 10^−3^ mol dm^−3^ in a solvent of hexane, and held there for 3 h. The target monolayer of APTES was thus formed on the hydroxylated silicon surface. After rinsing with Milli-Q water, the APTES-coated silicon substrates were put into the chamber of UV/O_3_ surface decontamination (Bioforce UV/Ozone ProCleaner Plus, USA) and irradiated for 1 h through a photomask consisting of stripes with ca. 50 μm width and a spacing of 30 μm. After completing UV irradiation, the samples were continually incubated with power off in an ozone environment for 15 min.

### 3.3 Deposition of Cu_*x*_S films

The copper precursor solution was prepared by dissolving CuSO_4_, EDTA and Na_2_S_2_O_3_ (mole ratio, 1:1:1) into Milli-Q water. The concentration of each constituent was adjusted to 10 mM. Droplets of diluted H_2_SO_4_ were slowly added to control the pH at ∼2.5. The patterned APTES-SAM wafers were then immersed in the fresh copper solution at 70 °C for about 2 h. Finally, the samples were taken out and washed with Milli-Q water by ultrasonication and dried with a stream of dry N_2_.

### 3.4 Characterization techniques

Optical microscopic images were collected on a charge-coupled-device (CCD) camera equipped to a microhardness tester (Axiophot, Germany). The structure and phase composition were characterized by grazing incidence X-ray diffraction (GIXRD) with Cu Kα radiation (40 kV, 40 mA) at an incidence angle of 1° (Panalytical, X’Pert Pro, Netherlands). A scanning probe microscope (Molecular Imaging, Agilent, USA) operated in contact mode was used to observe the patterning of the films and their morphology. Optical transmission studies were carried out using a UV–vis spectrophotometer (Varian, USA) in the wavelength range of 400–1000 nm. Scanning electrochemical microscopy (SECM) was carried out using a CHI-910B workstation (CH Instruments, Austin, USA) in the presence of 0.9 mM FcMeOH as a redox probe in 0.1 M KNO_3_ solution with a biased potential of 0.4 V. A three-electrode configuration was used for all amperometric detection with a 10 μm diameter ultramicroelectrode (UME) Pt tip (insulated in glass) as the working electrode, a Pt wire as the counter electrode, and Ag/AgCl (in 3 M KCl) as the reference electrode.

## 4 Results and discussion

### 4.1 The formation and surface characterization of patterned Cu_*x*_S films

**Fig. 2:**
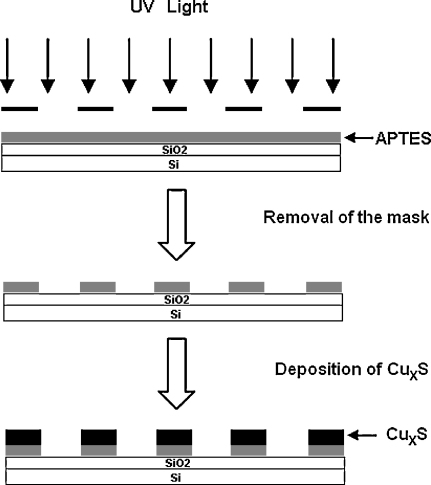
Schematic procedure for producing patterned Cu_*x*_S films on silicon substrates.

The strategy for the fabrication of patterned Cu_*x*_S thin films is illustrated in Fig. 2. The micro-pattern of the APTES substructure is formed through photolithography. The film is irradiated by UV light through the mask. In the exposed area, the UV Ozone etches the APTES layer and the bare SiO_2_/Si forms. The unexposed part does not undergo a decomposition reaction and therefore the APTES layer can be kept in the place, forming the desired micro-pattern (stripe lines). The patterned APTES substrate was then reacted with the precursor solution at 70 °C for a period of about 2 h. The copper sulfide particles deposit only on the APTES modified areas, so that a micro-pattern of Cu_*x*_S thin films is achieved. The function of the organic silicon compound is presumed to be as follows. The APTES molecules have NH_2_ groups which undergo partial protonation under the acidic conditions of pH 2.5, thus providing a positively charged surface. Since colloidal particles of copper sulfide have negative charge [18], they are absorbed on the APTES surface through attractive electrostatic interaction. On the other hand, the SiO_2_ surface is slightly negatively charged at the deposition pH which is just above the isoelectric point of pH 2 [35]. Thus, there is an electrostatic repulsion between Cu_*x*_S particles and SiO_2_ surface, which prevents deposition.

**Fig. 3:**
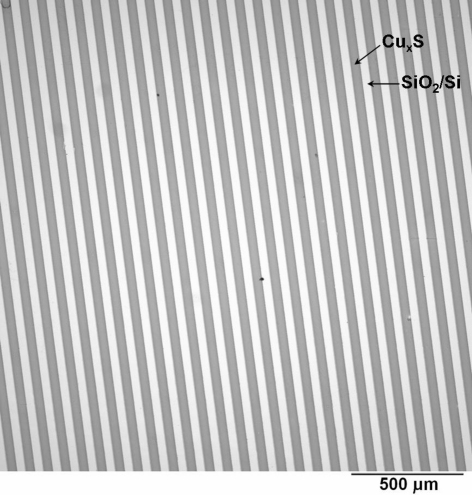
Optical microscope image of the patterned Cu_*x*_S film. The dark and bright stripes represent deposited Cu_*x*_S films and the bare silicon, respectively.

**Fig. 4:**
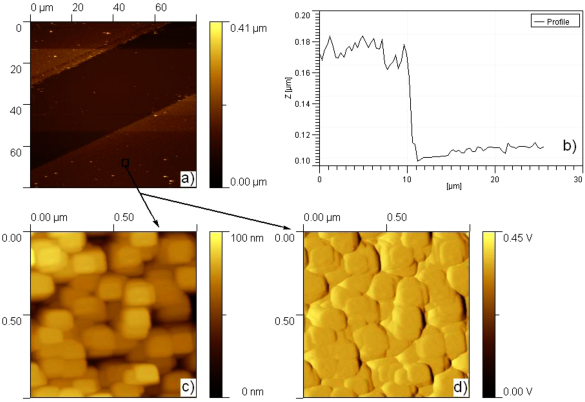
AFM image and section analysis of the patterned Cu_*x*_S film (a and b); (c) and (d) are the surface morphology and deflection image of as-deposited Cu_*x*_S film, respectively.

The optical image of copper sulfide patterns formed in this way is shown in Fig. 3. A sharp contrast between the patterned area and the SiO_2_/Si-passivated area is clearly demonstrated in the image. The deposition selectivity for Cu_*x*_S particles is high, and all of the APTES patterned area is occupied by the Cu_*x*_S film. Moreover, the width of the patterned Cu_*x*_S stripe lines is about 50 μm which is close to the dimension of the photomask (with ca. 50 μm width and a spacing of 30 μm). Fig. 4 gives the AFM images of the Cu_*x*_S patterned film on the silicon substrate. It shows only one stripe of the patterning in Fig. 4a because of the scanner size limitation. The brighter areas correspond to the higher regions (Fig. 4a). As can be clearly seen, the deposited thin film selectively and completely covers the APTES dominated areas. The thickness of the Cu_*x*_S film is about 80 nm (from the cross-section analysis (Fig. 4b)). The surface morphology of Cu_*x*_S film is also given at higher magnification in Fig. 4c and d. It can be seen that the deposited Cu_*x*_S film is densely packed and homogeneous. The Cu_*x*_S particles exhibit cubic crystal structure, and the particle size was about 125 nm after annealing for 2 h in argon atmosphere at 200 °C.

**Fig. 5:**
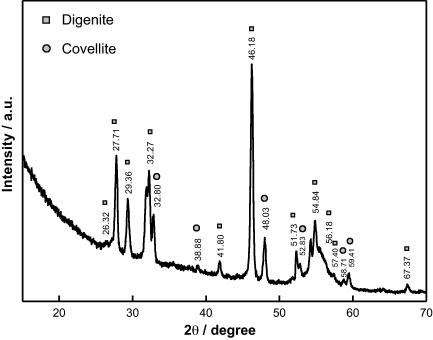
Grazing incidence XRD profile of the Cu_*x*_S thin film deposited on the APTES-modified silicon wafer. Two phases, hexagonal covellite (CuS) and cubic digenite (Cu_9_S_5_) are identified, and the dominant phase is digenite.

The GIXRD spectrum of the deposited Cu_*x*_S film is shown in Fig. 5. The peaks observed near 26.32°, 27.71°, 29.36°, 32.27°, 41.80°, 46.18°, 51.73°, 54.84°, 56.18°, 57.40° and 67.37° are closely associated with the standard Cu_9_S_5_ (digenite) phase [36]. The other peaks near 32.80°, 38.88°, 48.03°, 52.83°, 58.71° and 59.41° are associated with standard CuS (covellite) phase [36]. Thus it is concluded that the deposited film is a mixture of Cu_9_S_5_ and CuS phases.

### 4.2 Optical studies

**Fig. 6:**
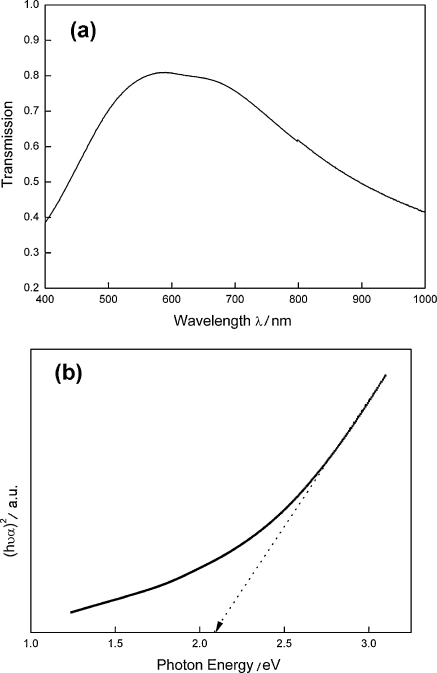
Optical transmission spectrum (a) and corresponding variation of absorption coefficient (*α*) with photon energy (*hν*) for the Cu_*x*_S film (b).

The optical transmission spectrum in the range of 400–1000 nm was recorded for the as-deposited copper sulfide film and is shown in Fig. 6a. The transmission is more remarkable around 550–700 nm in the visible region, while at the same time, a substantial decrease of transmission is found throughout the near-infrared region. The value of absorption coefficient *α* has been calculated by using the following equation [37]:

(4) $$\alpha =-\text{ln}\frac{T}{t}$$

where *T* is the measured transmittance value at a particular wavelength and *t* is the thickness of the film. The relationship between optical band gap energy (*E*_*g*_) and optical absorption coefficient (*α*) is determined by using the formula:

(5) $$\alpha h\nu =A{\left(h\nu -E_{g}\right)}^{n}$$

where *hν* is the photon energy and *A* is a constant which is related to the effective masses associated with valence and conduction bands [38]. For allowed direct transitions, *n* = 1/2 and allowed indirect transitions, *n* = 2. The experimental values of (*αhν*)^2^ against *hν* is plotted in Fig. 6b. The variation of (*αhν*)^2^ with *hν* is linear which indicates that a direct transition is present. Extrapolating the straight line portion of the plot of (*αhν*)^2^ against *hν* to the energy axis for zero absorption coefficient gives the optical band gap energy of the material. The optical band gap of Cu_*x*_S film estimated from Fig. 6b is 2.1 eV, which is comparable to the value of digenite copper sulfide thin film reported elsewhere [39,40]. Based on the optical band gap result and combined with X-ray diffraction analysis, we can conclude that the Cu_9_S_5_ (digenite) is the main phase in the as-prepared Cu_*x*_S film.

### 4.3 SECM characterization

SECM offers a unique means of visualizing surfaces by monitoring the limiting current at an ultramicroelectrode (UME) tip immersed in an electrolyte solution containing a suitable redox species [41]. The position of the UME is controlled by a micropositioner composed of three piezoelectric motors, which allow independent manipulation in the *x*, *y*, and *z* directions. Restricted diffusion at short tip-to-surface distances allows topological features to be mapped, and information related to the conductivity of the surface can be acquired from the analysis of feedback currents [42–44].

**Fig. 7:**
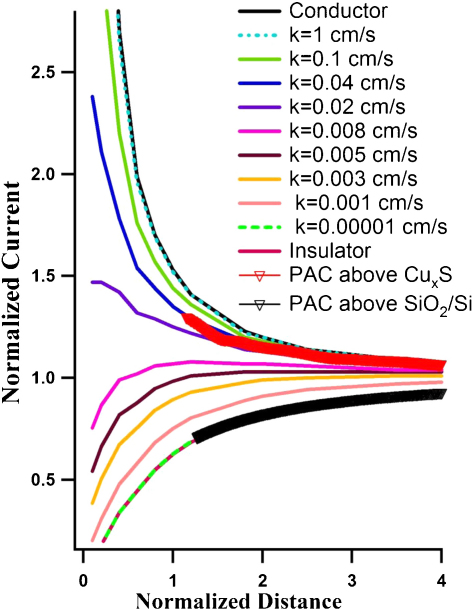
Probe approach curves (PACs) above the Cu_*x*_S substrate (red line with red triangles) and SiO_2_/Si substrate (black line with black triangles) recorded in 0.9 mM FcMeOH, 0.1 M KNO_3_ solution with 5 μm radius Pt disk at a biased potential of −0.4 V (*vs.* Ag/AgCl) superimposed with simulated PACs.

The PACs was plotted with normalized current (*I*, *I* = *i*_*T*_/*i*_*T*_,_∞_, *i*_*T*_ and *i*_*T*_,_∞_ are the tip currents measured at *L* and far from the substrate, respectively) *vs.* normalized distance (*L*, *L* = *d*/*a*, *d* is the tip-substrate distance and *a* is the UME tip radius). Fig. 7 shows PACs recorded above the infinite Cu_*x*_S surface (red line with red triangles) and infinite SiO_2_/Si surface (black line with black triangles), It can be seen that the Cu_*x*_S substrate has positive current feedback, while a decrease in the faradic current has been observed in SiO_2_/Si area. In addition, simulated PACs with the varied *k* value from 1 × 10^−5^ cm/s to 1 cm/s were demonstrated in Fig. 7. These simulated PAC results illustrate that the normalized current increase and the feedback switches from negative to positive feedback with the increased *k* value, i.e., the increased conductivity of the substrate. When the *k* value is 1 × 10^−5^ cm/s, the simulated PAC fits very well with the PAC above the insulator while the PAC with the *k* value of 1 cm/s is overlapped with the PAC for conductor. The electron transfer rate at the Cu_*x*_S substrate is determined to be 0.04 cm/s, which can be seen from Fig. 7 from the overlapped PAC for Cu_*x*_S substrate and the simulated PAC with a *k* value of 0.04 cm/s (blue curve). Ghilane et al. [45] studied the influence of surface modification of p-type silicon with different SAMs on interfacial electron transfer rate constant using SECM, and found that the bare p-type silicon substrate has an apparent electron-transfer rate constant *k* of 0.013 cm/s, while the apparent electron-transfer rate constant *k* decreases when the surface is coated by SAMs, in the order C_6_H_13_ (*k* = 0.0115 cm/s), C_10_H_21_ (*k* = 0.00082 cm/s), C_16_H_33_ (*k* = 0.00041 cm/s). Compared to the bare p-type silicon (without SiO_2_ layer), the Cu_*x*_S film possesses much better conductivity due to its high apparent electron-transfer rate constant, and probably exhibits semi-metallic properties.

**Fig. 8:**
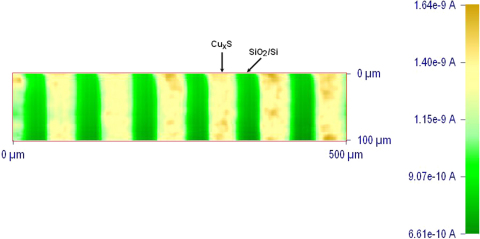
SECM image of a 500 μm × 100 μm area of micro-patterned Cu_*x*_S array in 0.9 mM FcMeOH, 0.1 M KNO_3_ solution and *E*_*tip*_ = 0.5 V (*vs.* Ag/AgCl).

Fig. 8 shows the SECM image (current map) of the micro-patterned Cu_*x*_S film obtained at a tip-sample distance around 15.0 μm. This distance was read from the simulated probe approach curve (the one that overlap the experimental probe approach curve above Cu_*x*_S substrate) with normalized current of 1.1 over the Cu_*x*_S band. The SECM image clearly shows the expected current regeneration contrast between the Cu_*x*_S and the SiO_2_/Si regions. It also reflects quite high edge acuity of the patterning. The yellow-colored areas indicate a faster charge transfer than that in the green-colored areas. The regions of high anodic current result from positive feedback due to the conductive Cu_*x*_S film. Notice that these regions surround rectangular arrays of low current, which are a consequence of negative feedback over the SiO_2_/Si. Both the size and the shape of the more conducting areas are similar to those of the Cu_*x*_S-containing regions evidenced by AFM and optical microscope. However, the SECM resolution being on the order of the UME probe radius (in our case, 5 μm), the fine structure of the Cu_*x*_S particles cannot be characterized by this technique, and consequently, the image shown in Fig. 8 provides a global view of the charge-transfer properties of the scanned surface.

## 5 Conclusions

In summary, we have presented a simple and convenient method for forming micro-patterned Cu_*x*_S films on a silicon surface. As the template for deposition, prepatterned APTES SAM was fabricated by UV-photolithography. Low-cost inorganic salts were used as the precursors. The films were prepared by chemical bath deposition in a relatively low acidity and environmentally benign solution. The Cu_*x*_S particles were selectively deposited on the SAM region, and not on the bare silicon area, due to the modification of surface charge by the APTES SAM. Optical microscope and AFM results showed that the Cu_*x*_S microstructure possessed a regular pattern and clear boundary. From optical adsorption the direct band gap of the resultant Cu_*x*_S film was found to be 2.1 eV. Combined with X-ray diffraction analysis, Cu_9_S_5_ (digenite) was shown to be the main phase in the Cu_*x*_S film. Electrochemistry studies using SECM have revealed that the Cu_*x*_S film had good electrical conductivity and the deposition selectivity of Cu_*x*_S was quite high. The apparent electron-transfer rate constant (*k*) in the micro-pattern of Cu_*x*_S dominated surface was estimated from SECM approach curve analysis and found to be 0.04 cm/s. This patterned Cu_*x*_S microstructure reveals the potential application of the p-type semi-metallic thin film in photovoltaic devices or nano/microscale electronics.
